# Efficacy and Safety of Lopinavir/Ritonavir for Treatment of COVID-19: A Systematic Review and Meta-Analysis

**DOI:** 10.3390/tropicalmed5040180

**Published:** 2020-11-28

**Authors:** Saad Alhumaid, Abbas Al Mutair, Zainab Al Alawi, Naif Alhmeed, Abdul Rehman Zia Zaidi, Mansour Tobaiqy

**Affiliations:** 1 Administration of Pharmaceutical Care, Ministry of Health, Al-Ahsa 31982, Saudi Arabia; 2 Research Center, Almoosa Specialist Hospital, Al-Ahsa 31982, Saudi Arabia; abbas.almutair@almoosahospital.com.sa; 3 Department of Pediatrics, College of Medicine, King Faisal University, Al-Ahsa 31982, Saudi Arabia; dr_z.alawi@hotmail.com; 4 Administration of Supply and Shared Services, Ministry of Health, Riyadh 11461, Saudi Arabia; nalhmeed@moh.gov.sa; 5 Research Center, Dr. Sulaiman Al Habib Medical Group, Riyadh 11461, Saudi Arabia; ar-zia@hotmail.com; 6 Department of Pharmacology, College of Medicine, University of Jeddah, Jeddah 21442, Saudi Arabia; mtobaiqy@uj.edu.sa

**Keywords:** COVID-19, efficacy, safety, kaletra, lopinavir/ritonavir, meta-analysis

## Abstract

(Background) Lopinavir-ritonavir (LPV/RTV) is a human immunodeficiency virus (HIV) antiviral combination that has been considered for the treatment of COVID-19 disease. (Aim) This systematic review aimed to assess the efficacy and safety of LPV/RTV in COVID-19 patients in the published research. (Methods) A protocol was developed based on the Preferred Reporting Items for Systematic reviews and Meta-Analysis (PRISMA) statement. Articles were selected for review from 8 electronic databases. This review evaluated the effects of LPV/RTV alone or in combination with standard care ± interferons/antiviral treatments compared to other therapies, regarding duration of hospital stay, risk of progressing to invasive mechanical, time to virological cure and body temperature normalization, cough relief, radiological progression, mortality and safety. (Results) A consensus was reached to select 32 articles for full-text screening; only 14 articles comprising 9036 patients were included in this study; and eight of these were included for meta-analysis. Most of these studies did not report positive clinical outcomes with LPV/RTV treatment. In terms of virological cure, three studies reported less time in days to achieve a virological cure for LPV/RTV arm relative to no antiviral treatment (−0.81 day; 95% confidence interval (CI), −4.44 to 2.81; *p* = 0.007, *I*^2^ = 80%). However, the overall effect was not significant (*p* = 0.66). When comparing the LPV/RTV arm to umifenovir arm, a favorable affect was observed for umifenovir arm, but not statically significant (*p* = 0.09). In terms of time to body normalization and cough relief, no favorable effects of LPV/RTV versus umifenovir were observed. The largest trials (RECOVERY and SOLIDARITY) have shown that LPV/RTV failed to reduce mortality, initiation of invasive mechanical ventilation or hospitalization duration. Adverse events were reported most frequently for LPV/RTV (*n* = 84) relative to other antivirals and no antiviral treatments. (Conclusions) This review did not reveal any significant advantage in efficacy of LPV/RTV for the treatment of COVID-19 over standard care, no antivirals or other antiviral treatments. This result might not reflect the actual evidence.

## 1. Introduction

Since the emergence of an unknown viral infection with its first cases in China in December 2019 and following the identification of this infection as 2019-new coronavirus disease (2019-nCoV, also known as COVID-19), caused by severe acute respiratory syndrome coronavirus 2 (SARS-CoV-2) [1], the world has worked to find effective therapeutics and vaccinations to treat hundreds of thousands of affected patients and to reduce the spread of this global pandemic [2].

As of 2 June 2020, there were 1104 registered clinical trials of COVID-19 therapeutics or vaccinations worldwide that either had ongoing or were recruiting patients; however, at that stage no drug or vaccine had officially been approved for COVID-19 [2,3]. These trials have produced mixed and conflicting results of positive or negative outcomes and inclusive evidence of efficacy or safety, that render the suspension of some trials inevitable, as in the hydroxychloroquine trials, which was suggested by the World Health Organization (WHO) in light of safety concerns [4]. This decision was reversed on 3 June 2020 [5], following a retraction of the research article by the Lancet as certain authors were not granted access to the underlying data [6]. As the pandemic evolves, the amount of evidence regarding the benefit of hydroxychloroquine in the treatment of COVID-19 patients has grown. A recent systematic review included 32 studies for a total 29,192 studied participants found treatment with hydroxychloroquine confers no benefit in terms of mortality in hospitalized patients with COVID-19 compared to standard care [7].

Lopinavir-ritonavir (LPV/RTV) is a protease inhibitor and nucleoside analog combination used for human immunodeficiency virus (HIV-1) and was also thought to be a potential treatment for COVID-19 [8], as its therapeutic value in the treatment of COVID-19 was assessed by in-vitro studies that claimed inhibition of several viral corona respiratory illnesses, including severe acute respiratory syndrome (SARS-CoV), and Middle East Respiratory Syndrome (MERS) [9,10,11]. Only recently, LPV/RTV therapy was hypothesized to be of no antiviral efficacy against SARS-CoV or MERS-CoV because the recommended dosages supplied to patients included in the published studies were subtherapeutic [12] and doses higher than 400 mg/100 mg twice daily are suggested [13].

Lopinavir (LPV) is an aspartic acid protease inhibitor of HIV, where inhibition of proteases enzymes is essential for the intervening of the viral infectious cycle. LPV is co-formulated with ritonavir (RTV) to boost the pharmacokinetic activity and half-life of LPV through the inhibition of cytochromes P450, providing adequate suppression of viral load and constant improvements in CD4+ cell counts, as demonstrated in randomized trials in naïve and experienced adult and child HIV patients [8].

There is conflicting evidence regarding the use of LPV/RTV for the treatment of COVID-19 patients; and evidence is currently scarce and of low quality. LPV/RTV is available as a single-tablet formulation (Kaletra^®^, North Chicago, IL, USA) in dosage strengths of 400/100 mg or 200/50 mg, and in clinical trials, this combination reduced rates of acute respiratory distress syndrome (ARDS) or death compared to supportive care or ribavirin alone in a matched cohort group during the early phase of viral acquisition [11].

LPV/RTV is being examined in several international clinical trials, including the RECOVERY trial and SOLIDARITY WHO trial [14], but did not gain authorization to be used emergently in the current pandemic in the USA by the Food and Drug Administration (FDA), which has approved only three pharmacologically different therapeutics for treatments of COVID-19, including antibiotic-hydroxychloroquine, immunotherapy-convalescent plasma therapy, and antiviral-remdesivir [2,14].

Among the clinical trials that did not find positive results for LPV/RTV, a study conducted by Bin Cao et al. published in the New England Journal of Medicine [15] revealed that treatment with LPV/RTV was not associated with clinical improvement beyond standard care or reduction in mortality rate at 28 days in hospitalized adult patients with severe COVID-19.

To date, LPV/RTV combination is available in some countries’ therapeutics guidelines including USA [16], Saudi Arabia [17], and Ireland [18], which means that the medicine has tenable evidence of efficacy; however, considering that early negative and conflicting results have emerged [15], there is a need to assess the efficacy and safety of this COVID-19 treatment in a systematic manner.

## 2. Aim of the Study

This systematic review and meta-analysis aimed to assess the efficacy and safety of LPV/RTV in COVID-19 patients in published research.

## 3. Methods

This systematic review was conducted with reference to the basics of Cochrane Handbook for Systematic Reviews of Interventions [19], described as stated by the Preferred Reporting Items for Systematic reviews and Meta-Analysis (PRISMA) statement [20].

### 3.1. Search Strategy and Selection Criteria

A systematic review protocol was developed based on PRISMA-P and the PRISMA statement. Published articles from 1 December 2019, to 20 November 2020, were selected for review from 8 electronic databases (PubMed, CINAHL, Embase, medRxiv, Proquest, Wiley online library, Medline, and Nature).

The focus of the review was LPV/RTV treatment in COVID-19 patients. The primary outcome was the efficacy of LPV/RTV in COVID-19 patients. The secondary outcome was adverse events associated with its use.

### 3.2. Inclusion Criteria

Readily accessible peer-reviewed full articles, observational cohort studies, and clinical trials were included.

### 3.3. Participants

Patients with a positive SARS-CoV-2 reverse-transcription polymerase chain reaction (RT-PCR) test of any age were included.

### 3.4. Intervention

The interventions were LPV/RTV alone or in combination with standard care ± interferons/antiviral treatments compared to other therapies.

### 3.5. Objectives

- Virological cure on day 7 after initiation of therapy (+ve to −ve polymerase chain reaction (PCR): non-detection of SARS-CoV-2 in nasopharyngeal swab).
- Clinical cure (time to body temperature normalization and time to cough relief).
- Radiological progression during drug treatment.
- Mortality at 28 days and death during treatment at any time.
- Safety and tolerability of lopinavir/ritonavir.

### 3.6. Comparisons

- lopinavir/ritonavir vs. no antiviral therapy (conventional therapy)/control.
- lopinavir/ritonavir in combination with other agents versus conventional therapy/control.

### 3.7. Searching Keywords

The search keywords included 2019-nCoV, 2019 novel coronavirus, COVID-19, coronavirus disease 2019, SARS-COV-2, lopinavir, ritonavir, combination, kaletra, treatment, efficacy, clinical trial, cohort, retrospective, and prospective.

### 3.8. Exclusion Criteria

Types of articles that were excluded included duplicate articles, editorials, reviews, case reports, and letters to editors.

Any research articles that did not include data on lopinavir/ritonavir use, did not include control patients’ group, or reported combined use of lopinavir/ritonavir with other antiviral medications were also excluded. Given the lack of clear benefit and potential for toxicity of hydroxychloroquine [21], studies with evidence on the benefit of LPV/RTV in combination with hydroxychloroquine use in hospitalized COVID-19 patients were excluded in our review.

### 3.9. Data Extraction and Analysis

Two reviewers (SA and MT) independently screened the titles with abstracts using the selection criteria. For relevant articles, full texts were obtained for further evaluation. Disagreements between the two reviewers after full text screening were reconciled via consensus by a third reviewer (AA) [22].

Inclusions and exclusions were recorded following PRISMA guidelines presented in the form of a PRISMA flow diagram and detailed reasons recorded for exclusion. Articles were categorized as clinical trials or cohort studies. The following data were extracted from the selected studies: authors; publication year; study location; study design and setting; sample size, age, and gender; details of study intervention and control therapies in addition to data on adverse events and treatment outcomes; time from symptom onset to treatment initiation; assessment of study risk of bias; and remarks on notable findings.

### 3.10. Risk of Biased Evaluation of Included Studies

The quality assessment of the studies was undertaken based on the revised Cochrane risk of bias tool (RoB 2.0) for randomized controlled studies [23]. The Risk of bias in non-randomized studies—of interventions (ROBINS-I) tool was used to assess non-randomized interventional studies [24], and the Newcastle Ottawa Scale for observational cohort studies [25]. Critical appraisal checklists appropriate to each study design were applied and checked by a third team member. 

Three investigators (SA, MT, and AA) separately evaluated the possibility of bias using these tools. Publication bias was not evaluated by funnel plot as there were only three studies that were included in the meta-analysis part of the study.

### 3.11. Assessment of Heterogeneity

Statistical heterogeneity was evaluated using the *χ*^2^ test and *I*^2^ statistics [19]. An *I*^2^ value of 0 to <40% was not considered as significant, 30% to 60% was regarded as moderate heterogeneity, 50% to 90% was considered substantial heterogeneity, and 75% to 100% was considered significant heterogeneity.

### 3.12. Statistical Analysis

Because all of the data were continuous and dichotomous data, either odds ratio (OR) or mean difference were used for estimating the point estimate, along with a 95% confidence interval (CI). In the absence of significant clinical heterogeneity, the meta-analysis using the Mantel Hazel method or inverse variance method for dichotomous data and continuous data were performed, respectively. Employing a conservative approach, a random effects model was used, which produces wider CIs than a fixed effect model. Review Manager (Version 5.3, Oxford, UK; The Cochrane Collaboration, 2014) was used to conduct all statistical analyses and generate forest plots.

## 4. Results

**Figure 1:**
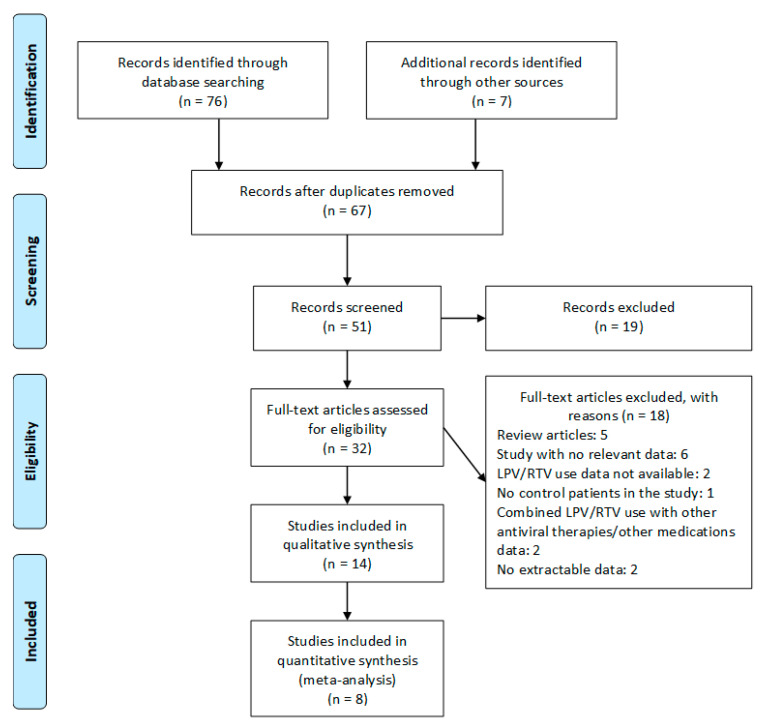
Preferred Reporting Items for Systematic reviews and Meta-Analysis (PRISMA) flow chart of the included studies. LPV/RTV, lopinavir/ritonavir.

A total of 8 literature databases were screened and 76 non-duplicate articles were identified, which were evaluated for possible inclusion using titles and abstracts. Out of these, 32 articles were selected for full-text screening and finally, 14 articles (total participants = 9036) were included in the systematic review, and eight articles were included in the meta-analysis; 18 articles were excluded following full-text screening (reasons: review = 5, study with no relative data = 6, LPV/RTV use data not available = 2, no control patients in the study = 1, combined LPV/RTV use with other antiviral therapies/other medications data = 2, no extractable data = 2). The PRISMA chart for the studies included is displayed in Figure 1. The details of the included studies are depicted in Table 1. Among these, two articles were in preprint versions [26,27].

### 4.1. Comparison 1: Efficacy and Safety of Lopinavir-Ritonavir (LPV/RTV) versus No Antiviral Therapy (Conventional Therapy) or Control

A total of eight studies [26,27,28,29,32,33,34,36] reported on LPV/RTV versus no antiviral therapy (conventional therapy) or control (*n* = 8405) in terms of efficacy and safety.

#### 4.1.1. Virological Cure on Day 7 Post-Initiation of Therapy (+ve to −ve PCR: Non-Detection of Severe Acute Respiratory Syndrome Coronavirus 2 (SARS-CoV-2) in Nasopharyngeal Swab)

##### LPV/RTV Versus No Antiviral Therapy (Conventional Cure): Virologic Cure at Day 7 Post-Initiation of Therapy

**Figure 2:**
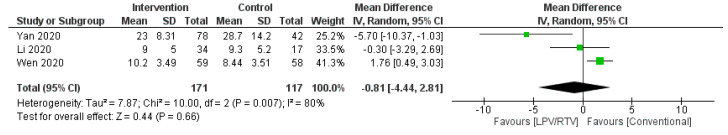
Time from +ve to −ve PCR (days) (LPV/RTV vs no antiviral treatment or conventional). CI, confidence interval; df, degrees of freedom; lopinavir/ritonavir (LPV/RTV).

Three studies reported on virological cure (*n* = 171 in LPV/RTV alone arm vs. *n* = 117 in conventional arm) on day 7 [27,32,34]. Significant mean difference was observed between the two arms in terms of virological cure (mean difference = −0.81 day; 95% CI, −4.44 to 2.81; *p* = 0.007, *I*^2^ = 80%; Figure 2).

##### LPV/RTV vs. Umifenovir: Virologic Cure at Day 7 Post-Initiation of Therapy

**Figure 3:**
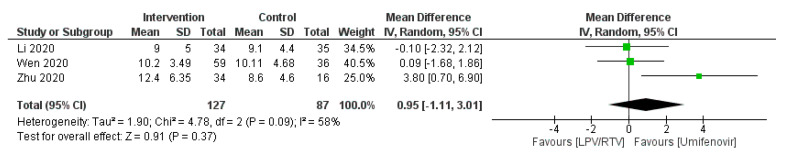
Time from +ve to −ve PCR (days) (LPV/RTV vs. umifenovir). CI, confidence interval; df, degrees of freedom; LPV/RTV, lopinavir/ritonavir.

Three studies reported on virological cure (*n* = 127 in LPV/RTV alone arm vs. *n* = 87 in umifenovir arm) on day 7 [27,32,36]. No significant mean difference was observed between the two arms in terms of virological cure (mean difference = 0.95 day; 95% CI, −1.11 to 3.01; *p* = 0.09, *I*^2^ = 58%; Figure 3).

##### LPV/RTV vs. Umifenovir Plus Lopinavir/Ritonavir: Virologic Cure at Day 7 Post-Initiation of Therapy

**Figure 4:**
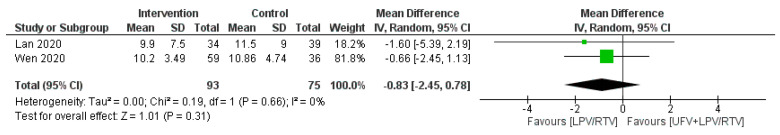
Time from +ve to−ve PCR (days) (LPV/RTV vs LPV/RTV plus umifenovir combination). CI, confidence interval; df, degrees of freedom; LPV/RTV, lopinavir/ritonavir; UFV, umifenovir.

Two studies reported on virological cure (*n* = 93 in LPV/RTV alone arm vs. *n* = 75 in umifenovir plus LPV/RTV arm) on day 7 [26,32]. No significant mean difference was observed between the two arms in terms of virological cure (mean difference = −0.83 day; 95% CI, −2.45 to 0.78; *p* = 0.66, *I*^2^ = 0%; Figure 4).

#### 4.1.2. Clinical Cure (Time to Body Temperature Normalization and Time to Cough Relief)

##### Time to Body Temperature Normalization

1. LPV/RTV vs. Umifenovir

**Figure 5:**
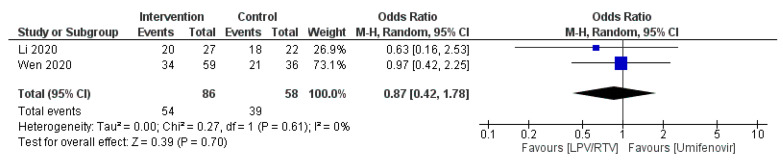
Time to body temperature normalization (days) (LPV/RTV vs umifenovir). CI, confidence interval; df, degrees of freedom; LPV/RTV, lopinavir/ritonavir.

Two studies reported on time to temperature normalization (*n* = 93 in LPV/RTV alone arm vs. *n* = 71 in umifenovir arm) [27,32]. No significant association was observed between the two arms in terms of temperature normalization (OR = 0.87 day; 95% CI, 0.42 to 1.78; *p* = 0.61, *I*^2^ = 0%; Figure 5).

2. LPV/RTV versus No Antiviral Therapy (Conventional)

**Figure 6:**
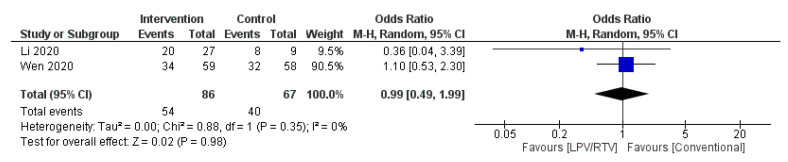
Time to body temperature normalization (days) (LPV/RTV vs. no antiviral treatment or conventional). CI, confidence interval; df, degrees of freedom; LPV/RTV, lopinavir/ritonavir.

Two studies reported on time to temperature normalization (*n* = 93 in LPV/RTV alone arm vs. *n* = 75 in conventional arm) [27,32]. No significant association was observed between the two arms in terms of temperature normalization (OR = 0.99 day; 95% CI, 0.49 to 1.99, *p =* 0.35, *I*^2^ = 0%; Figure 6).

##### Duration of Cough

1. LPV/RTV Versus Umifenovir: Rate of Cough Alleviation after 7 Days of Therapy

**Figure 7:**
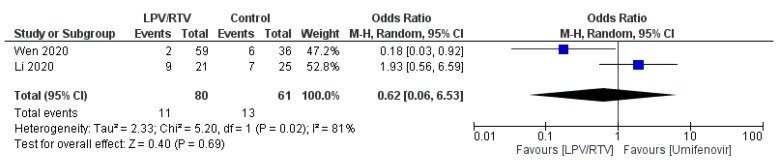
Rate of cough alleviation after 7 days of treatment (LPV/RTV vs. umifenovir). CI, confidence interval; df, degrees of freedom; LPV/RTV, lopinavir/ritonavir.

Two studies reported on cough alleviation (*n* = 93 in LPV/RTV alone arm vs. *n* = 71 in umifenovir arm) [27,32]. LPV/RTV alone arm had a significant lower number of cough days by 0.62 (95% CI 0.06 to 6.53, *p* = 0.02; *I*^2^ = 81%; Figure 7).

2. LPV/RTV vs. No Antiviral Therapy (Conventional): Rate of Cough Alleviation after 7 Days of Therapy

**Figure 8:**
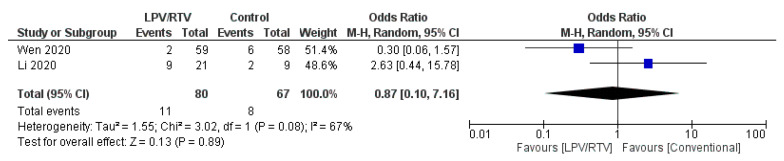
Rate of cough alleviation after 7 days of treatment (LPV/RTV vs. no antiviral treatment or conventional). CI, confidence interval; df, degrees of freedom; LPV/RTV, lopinavir/ritonavir.

Two studies reported on cough alleviation (*n* = 93 in LPV/RTV alone arm vs. *n* = 75 in conventional arm) [27,32]. No significant association was observed between the two arms in terms of cough alleviation (OR = 0.87 day; 95% CI, 0.10 to 7.16; *p* = 0.08, *I*^2^ = 67%; Figure 8).

#### 4.1.3. Radiological Progression during Drug Treatment

##### Rate of Improvement on Chest Computed Tomography (CT) after 7 Days of Treatment

1. LPV/RTV vs. Umifenovir

**Figure 9:**
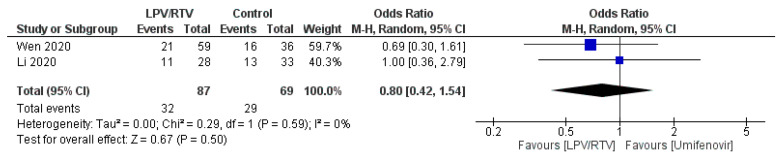
Rate of improvement on chest CT after 7 days of treatment (LPV/RTV vs. umifenovir). CI, confidence interval; df, degrees of freedom; LPV/RTV, lopinavir/ritonavir.

In terms of CT evidence for radiological progression of pneumonia/lung damage (*n* = 59 in the LPV/RTV arm vs. *n* = 71 in the umifenovir arm), treatment with LPV/RTV resulted in no significant decrease in the radiological progression (OR = 0.80; 95% CI, 0.42 to 1.54; *p* = 0.59, *I*^2^ = 81%; Figure 9) [27,32].

2. LPV/RTV vs. No Antiviral Therapy (Conventional)

**Figure 10:**
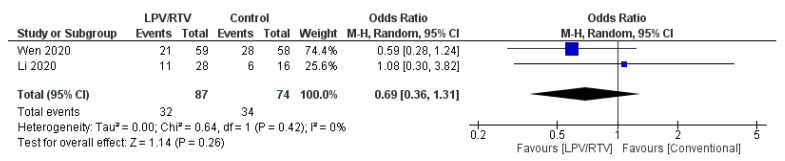
Rate of improvement on chest CT after 7 days of treatment (LPV/RTV vs. no antiviral treatment or conventional). CI, confidence interval; df, degrees of freedom; LPV/RTV, lopinavir/ritonavir.

In terms of CT evidence for radiological progression of pneumonia/lung damage (*n* = 71 in the LPV/RTV arm vs. *n* = 75 in conventional arm), treatment with LPV/RTV resulted in no significant decrease in the radiological progression (OR = 0.69; 95% CI, 0.36 to 1.31; *p* = 0.42, *I*^2^ = 0%; Figure 10) [27,32].

#### 4.1.4. Mortality at 28 Days and Death during Treatment at Any Time

##### Mortality at 28 Days

1. LPV/RTV vs. Standard of Care

**Figure 11:**
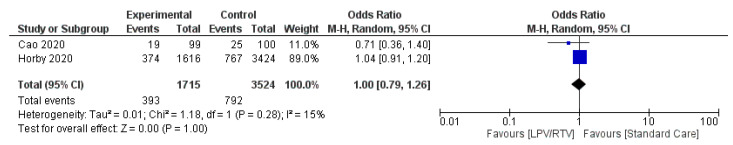
Rate of mortality at 28 days (LPV/RTV plus standard of care vs. standard of care alone). CI, confidence interval; df, degrees of freedom; LPV/RTV, lopinavir/ritonavir.

Two trials reported on mortality at 28 days (*n* = 1715 in LPV/RTV plus standard of care arm vs. *n* = 3524 in standard of care arm) [15,28]. No significant association was observed between the two arms in terms of mortality at 28 days (OR = 1.00; 95% CI, 0.79 to 1.26; *p* = 0.28, *I*^2^ = 15%; Figure 11).

##### Death during Treatment at Any Time

1. LPV/RTV vs. Standard of Care

**Figure 12:**
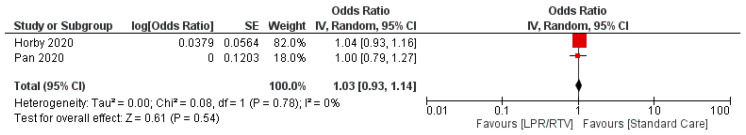
Rate of mortality at 28 days (LPV/RTV plus standard of care vs. standard of care alone). CI, confidence interval; df, degrees of freedom; LPV/RTV, lopinavir/ritonavir.

Two large trials reported on death during treatment at any time (*n* = 3015 in LPV/RTV plus standard of care arm vs. *n* = 4796 in standard of care arm) [28,29]. No significant association was observed between the two arms in terms of death during treatment at any time (OR = 1.03; 95% CI, 0.93 to 1.14; *p* = 0.78, *I*^2^ = 0%; Figure 12).

#### 4.1.5. Safety and Tolerability

##### Rate of Adverse Events of Treatment: LPV/RTV vs. Umifenovir

**Figure 13:**
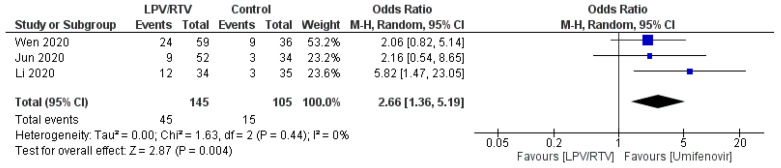
Rate of adverse events of treatment (LPV/RTV vs. umifenovir). CI, confidence interval; df, degrees of freedom; LPV/RTV, lopinavir/ritonavir.

A greater number of adverse events were reported in the LPV/RTV arms (*n* = 45) compared to the umifenovir groups (*n* = 14) (OR = 2.66; 95% CI, 1.36 to 5.19; *p* = 0.44, *I*^2^ = 0%; Figure 13) [27,32,33].

##### Rate of Adverse Events of Treatment: LPV/RTV vs. No Antiviral Treatment (Conventional)

**Figure 14:**
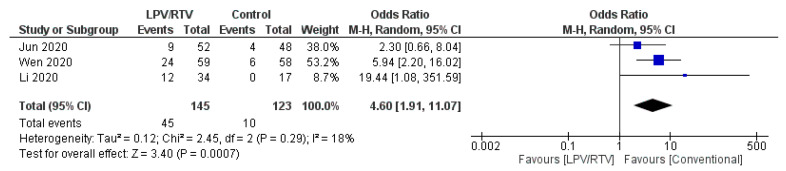
Rate of adverse events of treatment (LPV/RTV vs. no antiviral treatment or conventional). CI, confidence interval; df, degrees of freedom; LPV/RTV, lopinavir/ritonavir.

A greater number of adverse events were reported in the LPV/RTV arms (*n* = 45) compared to the no antiviral treatment or conventional arms (*n* = 10) (OR = 4.6; 95% CI, 1.91 to 11.07; *p* = 0.29, *I*^2^ = 18%; Figure 14) [27,32,33].

### 4.2. Comparison 2: Efficacy and Safety of LPV/RTV along in Combination with Other Agents versus No Antiviral Therapy (Conventional Therapy) or Control

A total of four studies evaluated the efficacy of LPV/RTV plus interferon (IFN) [30,31,35,37] and three studies [30,31,37] evaluated the safety of the combination. Other studies evaluated the efficacy of LPV/RTV plus standard care [15,28], ribavirin [31], or umifenovir [26,32,37], and evaluated the safety of these combinations. 

In terms of the efficacy of the combination in patients with COVID-19, LPV/RTV plus IFN combination in addition to ribavirin was safe and superior to LPV/RTV alone by shortening the median time from the start of study treatment to negative nasopharyngeal swab (7 days [IQR 5–11]) compared to the LPV/RTV arm (12 days [IQR 8–15]; hazard ratio 4.37 [95% CI 1.86–10.24], *p* = 0.001) [31]. Additionally, combination treatment with LPV/RTV plus IFN and umifenovir had a more evident therapeutic effect in a shorter time by normalizing body temperature (4.8 ± 1.94 days vs. 7.3 ± 1.53 days, *p* = 0.03) and turning PCRs negative (7.8 ± 3.09 days vs. 12.0 ± 0.82 days, *p* = 0.02) compared to the umifenovir plus IFN arm with no evident toxic and side effects [37]. However, the use of LPV/RTV plus IFN combination resulted in fewer therapeutic responses on COVID-19 in terms of viral clearance [median (interquartile range, IQR), 4 (2.5–9) d versus 11 (8–13) d, *p* < 0.001) and chest CT changes (91.43% vs. 62.22%), *p* = 0.004] compared to the favipiravir plus IFN combination. Favipiravir arm patients had fewer adverse events (AEs) compared to the LPV/RTV arm (11.43% vs. 55.56%) (*p* < 0.001) [30]. Additionally, no significant difference in average PCR negative conversion times among IFN plus LPV/RTV or IFN plus LPV/RTV plus ribavirin treatment arms [35]. In another cohort study, more patients turned SARS-CoV-2 PCR negative in the LPV/RTV plus umifenovir combination group compared to the LPV/RTV monotherapy group (after 7 days: 75% vs. 35% of patients were PCR negative in the combination therapy and monotherapy, respectively, *p* < 0.05; and after 14 days: 94% vs. 52.9% of patients were PCR negative in the combination therapy and monotherapy, respectively, *p* < 0.05) [38]. Moreover, chest CT scans were improving for 69% of patients in the combination group after seven days, compared with 29% in the monotherapy group (*p* < 0.05) [38].

The combination of LPV/RTV, in addition to standard care, or standard care alone exhibited no difference in the time to clinical improvement (hazard ratio for clinical improvement, 1.31; 95% CI, 0.95 to 1.80) with similar 28-day mortality (19.2% vs. 25.0%; difference, −5.8 percentage points; 95% CI, −17.3 to 5.7) [15]. In another recent large study, LPV/RTV combined with standard care was not associated with reductions in 28-day mortality, duration of hospital stay, or risk of progressing to invasive mechanical ventilation or death [28].

## 5. Discussion

This systematic review included 14 articles relating to the efficacy and safety of LPV/RTV in COVID-19 patients, with a total of 9036 patients included, and only eight articles, that comprised 8438 patients had findings on the efficacy and safety of LPV/RTV alone or in combination with standard care ± interferons/antiviral treatments compared to other therapies in the treatment of COVID-19, were deemed legible for quantitative synthesis (meta-analysis) [26,27,28,29,32,33,34,36].

In terms of virological cure, three studies reported less time in days for LPV/RTV arm (*n* = 171) compared with no antiviral therapy (conventional) (*n* = 117); however, the overall effect was not significant (mean difference = −0.81 day; 95% CI, −4.44 to 2.81; *p* = 0.66), similarly the virological cure for LPV/RTV alone (*n* = 127) versus the umifenovir arm (*n* = 87) (*p* = 0.37), or LPV/RTV versus umifenovir plus LPV/RTV (*p* = 0.31) [26,27,32,33,34,36].

Two studies reported no significant effect on time to temperature normalization for LPV/RTV arm (*n* = 93) versus umifenovir arm (*n* = 71) (OR = 0.87 day; 95% CI, 0.42 to 1.78; *p* = 0.70, *I*^2^ = 0%); or alleviation of cough duration (*p* = 0.69) [27,32]. The total number of cough days was found to be lower in the LPV/RTV arm compared with the umifenovir arm or no antiviral therapy (conventional) arm after 7 days of treatment; however, the overall effect was found to be not significant [27,32]. Although a favorable therapeutic effect for umifenovir was observed in a small cohort study when the drug was combined with LPV/RTV treatment in (*n* = 16) COVID-19 patients rather than LPV/RTV alone (*n* = 17) [38]; it should be noted that the treatment of LPV/RTV alone groups (*n* = 127) versus umifenovir plus LPV/RTV groups (*n* = 69) did not reveal any significant mean difference between the two groups in terms of virological cure at day seven [26,32,37]. In another study that involved 81 COVID-19 patients, the umifenovir treatment group had a longer hospital stay than patients in the control group (13 days (IQR 9–17) vs. 11 days (IQR 9–14), *p* = 0.04) [39]. Of note, umifenovir, which is branded as Arbidol^®^, has a wide antiviral activity against RNA and DNA viruses, is licensed in Russia and China for the treatment and prophylaxis of influenza and recommended for treatment of MERS-CoV, was investigated in SARS-CoV, and is currently being trialed in COVID-19 patients [40].

In terms of CT evidence for radiological progression of pneumonia/lung damage, fewer patients exhibited radiological progression in the LPV/RTV arm compared with the umifenovir arm or no antiviral therapy (conventional) arm after 7 days of treatment, this effect was not significant (*p* = 0.59), and similarly, with LPV/RTV (*n* = 71) versus no antiviral therapy [27,32].

It is worth mentioning that initiating therapy earlier is known to be more effective [41], since systemic hyperinflammation rather than viral pathogenicity dominates later stages of SARS-CoV-2 infection. Although patients in five of the studies [15,27,30,31,34] included in our review were administered LPV/RTV early in the infection (median of <7 days); LPV/RTV therapy was not found to be totally effective.

In terms of safety, this study found greater adverse events reported in the LPV/RTV arm versus no antiviral treatment (conventional) or umifenovir, respectively. Adverse events associated with LPV/RTV alone or in combination with other medicines were reported in COVID-19 patients, and were typically gastrointestinal (GIT) in nature, including nausea, vomiting, and diarrhea [32]; nevertheless, serious GIT adverse drug reactions such as acute gastritis and GIT bleeding and acute kidney injury (*n* = 3) were also reported [32]. It was reported that most ADRs associated with LPV/RTV in combined groups of medicines are resolved within three days of drug initiation [30].

To address the efficacy and safety of LPV/RTV combined with other drugs in patients with COVID-9, LPV/RTV plus IFN combination in addition to ribavirin was found to be superior and safer than LPV/RTV alone by shortening the time to negative nasopharyngeal swab compared to the LPV/RTV arm alone [31]. Additionally, a combined treatment regimen of LPV/RTV plus IFN and umifenovir resulted in a shorter time by normalizing body temperature and turning PCRs negative compared to the umifenovir plus IFN arm with reasonable safety profile [37]. However, the use of LPV/RTV plus IFN combination resulted in less therapeutic responses on COVID-19 in terms of viral clearance and chest CT changes compared to the favipiravir plus IFN combination. Favipiravir arm patients had fewer AEs than patients in the LPV/RTV arm [30]. Additionally, there was no significant difference in average PCR negative conversion times among IFN plus LPV/RTV or IFN plus LPV/RTV plus ribavirin treatment arms [35]. The combination of LPV/RTV, in addition to standard care, or standard care alone revealed no difference in the time to clinical improvement, duration of hospitalization, initiation of invasive mechanical ventilation and death [15,28,29]. A serious case of elevated alanine aminotransferase (ALT) was reported [28], GI AEs were more common in the LPV/RTV group and treatment was stopped early in 13.8% because of AEs [15].

In a recent systematic review that included 41 studies which considered therapeutics for COVID-19, LPV/RTV was found to be the third therapy associated with positive outcomes (54.9%) with less negative outcomes (12.3%) compared to systemic corticosteroids (21.3%), remdesivir (16.9%), moxifloxacin (13.4%) and oseltamivir (12.5%) [2]; however, further controlled studies were needed to draw a valid conclusion. Antiviral ineffectiveness of LPV/RTV against SARS-CoV-2 in the studies included in our review was justified by the necessity to give the drug at a daily amount higher than 800 mg/200 mg; as an in vitro analysis identified antiviral activity of LPV/RTV against SARS-CoV-2 with a half-maximal effective concentration (EC_50_) of 16.4 μg/mL [42]. However, there is a potential to intoxicate the patient, because high doses of LPV/RTV may lead to delayed ventricular repolarisation (QT prolongation) [7]. Thus, it might be logical to argue that there is a need to determine the effective and safe dose of LPV/RTV against the SARS-CoV-2 virus for better clinical benefit [13].

It is important to consider drug concentrations at the site of infection, and currently, the lack of robust lung penetration data is an important gap that exists for many agents being considered for repurposing. In the case of LPV/RTV, lung penetration is complex and not well understood; however, typically it is the plasma-free fraction that is available to penetrate into tissues. Therefore, given its potency, lung penetration of LPV/RTV would have to be high to provide concentrations in the therapeutic range [43]. The antiviral activity in vivo is estimated by calculating the ratio of unbound drug concentrations achieved in the lung at the administered dose to the in vitro EC50 (R_LTEC_) [44]. Even though the majority of the observed total LPV/RTV plasma concentrations in COVID-19 patients were above the published EC50 for SARS-CoV-2 [42], boosted LPV/RTV is unlikely to attain sufficient effective levels in the lung to inhibit the virus. Indeed, the largest trials of RECOVERY [28] and SOLIDARITY [29] found LPV/RTV had little or no effect on overall mortality, initiation of ventilation and duration of hospital stay in hospitalized patients. 

There is uncertainty about the optimal approach to treat hospitalized COVID-19 patients. Management approaches are based on limited data and evolves rapidly as clinical data emerge. For patients with non-severe disease, care is primarily supportive, with close monitoring for disease progression. Remdesivir is suggested in hospitalized patients with severe disease (i.e., they have hypoxia) but who are not yet on oxygen [45,46]. For patients who are receiving supplemental oxygen (including those who are on high-flow oxygen and noninvasive ventilation), low-dose dexamethasone and, if available, remdesivir is/are suggested [47,48]. However, the optimal role of remdesivir remains uncertain, and some guidelines panels (including the WHO) suggest not using it in hospitalized patients because there is no clear evidence that it improves patient-important outcomes for hospitalized patients (e.g., mortality, need for mechanical ventilation). In general, use of LPV/RTV for treatment of SARS-CoV-2 in hospitalized patients is not suggested as several trials have failed to prove efficacy [15,28,29]. Evidence as to whether LPV/RTV is beneficial in outpatients with mild or moderate severity COVID-19 infection is lacking; therefore, use of LPV/RTV is suggested in outpatients only in the context of a clinical trial.

Vaccines to prevent COVID-19 infection are considered the most promising approach for controlling the pandemic. COVID-19 vaccine development is occurring at an unprecedented pace. Several different platforms are being utilized to develop COVID-19 vaccines such as: inactivated virus or live-attenuated virus platforms (traditional methods); recombinant proteins and vector vaccines (newer methods); and RNA and DNA vaccines (methods never previously employed in a licensed vaccine) [49]. Several vaccine candidates have demonstrated immunogenicity without major safety concerns in early-phase human trials [50]. Two mRNA vaccine candidates have also been reported to have approximately 95% vaccine efficacy [51,52]. AstraZeneca’s Oxford coronavirus vaccine is 70% effective on average, data shows, with no safety concerns [53]. Importantly, the AstraZeneca vaccine can be distributed and administered within existing healthcare systems, as it can be stored, transported and handled in normal refrigerated conditions for at least six months, the company said. The vaccine will also be cheaper than rival coronavirus vaccines from makers Pfizer and Moderna [53].

Since disease resulting from SARS-CoV infection is driven by both virus and host immune response factors, depending on the stage of the disease progression, early initiation of antiviral therapy, and/or holistic combination therapies will likely be needed to diminish virus replication, immunopathology, and/or promote repair and restoration of pulmonary homeostasis [54]. Until sufficient evidence is available, the WHO has warned against physicians and medical associations recommending or administering unproven treatments to patients with SARS-CoV-2 or people self-medicating with them.

The key limitations of this study were the limited number of clinical studies investigating the efficacy and safety of LPV/RTV in combination with a limited number of participants. Another limitation is the inability to perform any type of meta-analysis specifically for the results of efficacy and safety of using LPV/RTV in combination with other agents versus no antiviral therapy (conventional therapy) or control because of the large methodological differences. Despite these limitations, this systematic review provided valuable insight into the efficacy, safety, and clinical outcomes of LPV/RTV alone or with other antiviral medications.

## 6. Conclusions

The small number of studies included in this systematic review and meta-analysis study did not reveal any statistically significant advantage in the efficacy of LPV/RTV in COVID-19 patients, over no antiviral or other antiviral treatments. In terms of safety, this study found a greater number of adverse events reported in LPV/RTV arm versus no antiviral treatment (conventional) or umifenovir arms, respectively. There is a general understanding of the need to conduct large randomized clinical trials to determine the efficacy and safety of LPV/RTV in the treatment of COVID-19. Ideally, these studies should be double-blinded and conducted in a wide range of settings.

## Figures and Tables

**Table 1:** Data extracted from included papers (*n* = 14).

Author, Year [Reference] from, Study Location	Study Design and Setting	Age (Year)	Male, *n* (%)	Population	Intervention	Control	Time from Symptom Onset to Treatment Initiation	Outcome	AEs in LPV/RTV and Control Arm	Assessment of Study Risk of Bias (Tool Used; Finding)	Remark
Horby et al. 2020 [28]; United Kingdom	Randomized open-label controlled trial; multicenter	Mean (SD), 66.3 (15.9)	3077 (61.1)	Clinically suspected or laboratory confirmed SARS-CoV-2 infection cases of any age Consistent characteristics across groups for age, sex, ethnicity, duration of symptoms before randomisation, amount of respiratory support at randomisation, and baseline predicted risk of death	1616 patients received: LPV/RTV (oral): 400 mg/100 mg twice daily for 10 days or until discharge, if sooner PLUS standard care * for 10 days or until discharge, if sooner	3424 patients received: Standard care alone * for 10 days or until discharge, if sooner	Not reported	Mortality at 28 days: 23% patients allocated to LPV/RTV and 22% patients allocated to usual care died within 28 days (RR 1.03, 95% CI 0.91–1.17; *p* = 0.60) Time until discharge alive from hospital: median 11 days [IQR 5 to >28] in both groups Patients discharged from hospital alive within 28 days: (RR 0.98, 95% CI 0.91–1.05; *p* = 0.53) Patients met the endpoint of invasive mechanical ventilation (RR 1.15, 95% CI 0.95–1.39; *p* = 0.15); or death (RR 1.04, 95% CI 0.93–1.16; *p* = 0.54)	In the LPV/RTV group, there was a serious case of elevated ALT that did not meet standard criteria for drug-induced liver injury Detailed information on non-serious adverse reactions or reasons for stopping treatment were not collected	RoB 2, low risk of bias	LPV/RTV was not associated with reductions in 28-day mortality, duration of hospital stay, or risk of progressing to invasive mechanical ventilation or death Since preliminary results of RECOVERY trial were made public, WHO has halted the LPV/RTV monotherapy and the LPV/RTV plus IFN-b combination groups of the SOLIDARITY trial
Pan et al. 2020 [29]; Multi-country	Randomized open-label controlled trial; multicenter	<50 years: 36.5% 50–69 years: 7% 70+ years: 20.8%	1653 (59.6)	Hospitalized confirmed COVID-19 cases aged ≥18 years and not known to have received any study drug Patient characteristics were well balanced between the two groups	1399 patients received: LPV/RTV (oral): 400 mg/100 mg twice daily for 10 days	1372 patients received: standard care *	Not reported	Death (with 95% CIs and numbers dead/randomized, LPV/RTV vs. its control) was: RR 1.00 (0.79–1.25, *p* = 0.97; 148/1399 vs. 146/1372) Initiation of ventilation: 124 (LPV/RTV) vs. 119 (control) Patients still hospitalized at day 7: 68% (LPV/RTV) vs. 59% (control)	No death was attributed to LPV/RTV due to renal or hepatic disease	RoB 2, low risk of bias	LPV/RTV did not reduce mortality (in unventilated patients or any other subgroup of entry characteristics), initiation of ventilation or hospitalization duration
Cai et al. 2020 [30]; China	Nonrandomized open-label controlled trial; single center	Median (IQR), 47 (35.7–61)	35 (43.8)	Confirmed COVID-19 cases aged 16–75 years No significant differences between the baseline characteristics of the two arms FPV treated patients were older (43 years) compared with LPV/RTV arm (49 years) All patients were moderate cases as defined by NHC [1]	45 patients received: LPV/RTV (oral): 400 mg/100 mg twice daily on days 1–14 PLUS IFN-α1b (aerosol inhalation): 5 million IUs twice daily	35 patients received: FPV (oral): 1600 mg twice daily on Day 1 and 600 mg twice daily on days 2–14 PLUS IFN-α1b (aerosol inhalation): 5 million IUs twice daily	Less than 7 days	Viral clearance: shorter viral clearance time for FPV arm (median (IQR), 4 (2.5–9) days versus 11 (8–13) days, *p* < 0.001) Chest CT changes: more imaging improvement rate in FPV arm (91.43% vs. 62.22%), *p* = 0.004	FPV arm patients had less AEs compared to the LPV/RTV group (11.43% vs. 55.56%) (*p* < 0.001) Two patients had diarrhea, one had a liver injury, and one had a poor diet in the FPV arm There were five patients with diarrhea, five with vomiting, six with nausea, four with rash, three with liver injury, and two with chest tightness and palpitations in the LPV/RTV arm	ROBINS-I, moderate risk of bias	Two patients in the FPV group turned negative for viral RNA detection in nasopharyngeal swabs at days 18 and 21 For patients in the LPV/RTV group, the viral RNA detection all turned negative within 27 days
Cao et al. 2020 [15]; China	Randomized open-label controlled trial; single center	Median (IQR), 58 (49–68)	120 (60.3)	Confirmed COVID-19, having a SaO_2_ of 94% or less or a ratio of the PaO_2_ to the FiO_2_ of less than 300 mmHg No important between-group differences in demographic characteristics, baseline laboratory test results, distribution of ordinal scale scores, or NEWS2 scores at enrollment	99 patients received: LPV/RTV (oral): 400/100 mg twice daily PLUS standard care * for 14 days	100 patients received: standard care * alone for 14 days	13 days (IQR, 11 to 16 days)	Time to clinical improvement: no difference in the time to clinical improvement for patients in the LPV/RTV group and the standard-care * group (HR for clinical improvement, 1.31; 95% CI, 0.95 to 1.80) Mortality at 28 days was similar in the two groups (19.2% vs. 25.0%; difference, −5.8 percentage points; 95% CI, −17.3 to 5.7) Percentages of patients with detectable viral RNA at various time points were similar LPV/RTV led to a median time to clinical improvement that was shorter by 1 day than that observed with standard care* (HR, 1.39; 95% CI, 1.00 to 1.91)	GI AEs were more common in the LPV/RTV group, but serious AEs were more common in the standard care * group. LPV/RTV treatment was stopped early in 13.8% because of AEs	RoB 2, low risk of bias	Most patients were severely unwell and required urgent clinical attention Systemic glucocorticoids were administered (33.0% in patients of LPV/RTV group and 35.7% in patients of standard-care * alone group)
Hung et al. 2020 [31]; Hong Kong	Randomized open-label trial; multicenter	Median (IQR), 52 (32–62)	68 (54)	Confirmed COVID-19 cases and aged at least 18 years, a NEWS2 of at least 1, and symptom duration of 14 days or less upon recruitment. Age, sex, and baseline demographics in each group were similar	41 patients received: LPV/RTV (oral): 400/100 mg twice daily (control group) for 14 days	86 patients received: LPV/RTV (oral): 400/100 mg twice daily PLUS Ribavirin (oral): 400 twice daily PLUS IFN-beta-1b (SCI): three doses of 8 million IUs of interferon beta-1b on alternate days (combination group); for 14 days	5 days (IQR 3–7)	Combination group had a significantly shorter median time from start of study treatment to negative nasopharyngeal swab (7 days [IQR 5–11]) than the LPV/RTV group (12 days [8,9,10,11,12,13,14,15]; HR 4.37 [95% CI 1.86–10.24], *p* = 0.0010)	AEs included nausea and diarrhea with no difference between the two groups. One patient in the control group discontinued LPV/RTV because of biochemical hepatitis	RoB 2, some concerns risk of bias	No patients died during the study
Li et al. 2020 [27]; China	Randomized blinded trial; single center	Mean (SD), 49.4 (14.7)	40 (46.5)	Mild/moderate confirmed COVID-19 cases aged 18–80 years Baseline characteristics of the three groups were comparable	34 patients received: LPV/RTV (oral): 200/50 mg twice daily for 7–14 days	35 patients received: Umifenovir (oral): 200 mg three times daily for 7–14 days OR 17 patients received no antiviral therapy (conventional)	3.5 days (IQR, 2 to 6 days)	Rate of positive-to-negative conversion of SARS-CoV-2 nucleic acid was similar between groups (all *p* > 0.05) There were no differences between groups in the rates of antipyresis, cough alleviation, or improvement of chest CT at day 7 or 14 (all *p* > 0.05) At day 7, 23.5% patients in the LPV/RTV group, 8.6% in the umifenovir group, and 11.8% in the control group showed a deterioration in clinical status from moderate to severe/critical (*p* = 0.206)	Overall, 35.3% of patients in the LPV/RTV group and 14.3% in the umifenovir group experienced AEs No apparent AEs occurred in the control group	RoB 2, high risk of bias	Study was blinded to participants, physicians, and radiologists who reviewed data but open label to clinicians who recruited patients and research staff All three groups were treated with Standard care * if in need
Lan et al. 2020 [26]; China	Retrospective; cohort; multicenter	Mean (SD), 55.8 (15.2)	37 (50.7)	Confirmed COVID-19 cases treated with LPV/RTV alone or combined with umifenovir Different age, sex, and baseline demographics in each group	34 patients received: LPV/RTV (oral): 400/100 mg twice daily for 14 days	39 patients received: LPV/RTV (oral): 400/100 mg twice daily PLUS Umifenovir (oral): 200 mg three times daily; at least for 3 days	Not reported	Treatment with LPV/RTV alone was not different from LPV/RTV combined with umifenovir in overall cure rate (92.3% and 97.1%, respectively) LPV/RTV combined with umifenovir led to a median time of hospital stay that was shorter by 1.5 days (12.5 days vs. 14 days) COVID-19 RNA clearance was 92.3% in LPV/RTV and 97.1% in combination therapies group Mean time of virus turning negative was 11.5 ± 9.0 days in combination group compared to 9.9 ± 7.5 in single therapy group	Not reported	NOS, 5	All eligible patients received standard care * if necessary
Wen et al. 2020 [32]; China	Retrospective; cohort; single center	Mean (SD), 49.9 (16.1)	81 (45.5)	Confirmed COVID-19 cases aged ≥18 years with a hospital stay longer than 14 days No statistically significant difference in baseline characteristics before treatment between patients in LPV/RTV group, umifenovir group, combination (LPV/RTV and umifenovir) group and conventional treatment (no antiviral therapy) group	59 patients received: LPV/RTV (oral): 200/50 mg twice daily for 7 days	36 patients received: Umifenovir (oral): 200 mg three times daily for 7 days OR 25 patients received: Combined antiviral therapies (LPV/RTV AND umifenovir; same dosages for 7 days) OR 58 patients received no antiviral therapy (conventional group)	Not reported	Time for pharyngeal swab PCR to turn negative was (10.20 ± 3.49 days) in LPV/RTV group, (10.11 ± 4.68 days) in umifenovir group, (10.86 ± 4.74 days) in LPV/RTV plus umifenovir group, and (8.44 ± 3.51 days) in conventional group No significant difference in the rate of nasopharyngeal swab new coronavirus nucleic acid conversion, clinical symptom improvement rate, and lung infection imaging improvement rate (*p* > 0.05). There was a statistically significant difference in the ratio of normal/mild to severe/critically severe on the 7th day in the four groups (χ^2^ = 9.311, *p* = 0.017): the combined group (24.0%), umifenovir group (16.7%), LPV/RTV group (5.4%), conventional treatment group (5.2%)	AEs in the three groups of patients using antiviral drugs was significantly higher than that in the conventional treatment group (χ^2^ = 14.875, *p* = 0.002)	NOS, 5	All three groups were treated with standard care * if in need
Jun et al. 2020 [33]; China	Retrospective; cohort; single center	Median (IQR), 48 (35–62)	69 (51.5)	Confirmed COVID-19 cases No statistically significant differences in the demographic data, clinical manifestations, laboratory examinations, and chest CT examination of patients in the LPV/RTV group, umifenovir group, and control (no antiviral therapy) group (all *p* > 0.05)	52 patients received: LPV/RTV (oral): 200/50 mg twice daily for 5 days	34 patients received: Umifenovir (oral): 200 mg three times daily for 5 days OR 48 patients received no antiviral therapy (conventional group)	Not reported	Median time for the body temperature to return to normal in the umifenovir group and the LPV/RTV group was 6 days, and the conventional group was 4 days (χ^2^ = 2.37, *p* = 0.31). Median time of viral nucleic acid negative in respiratory tract specimens of the three groups was 7 days after treatment. Viral nucleic acid negative in the LPV/RTV group was 71.8% and 82.6% in the umifenovir group, the conventional group was 77.1% (χ^2^ = 0.46, *p* = 0.79) 42.3% patients in the LPV/RTV group, 35.3% patients in the umifenovir group, and 52.1% patients in the conventional group still had progressive imaging on the 7th day after treatment (χ^2^ = 2.38, *p* = 0.30)	17.3% in the LPV/RTV group had AEs, including nausea, diarrhea, and other GI symptoms; 8.8% in the umifenovir group had AEs, including diarrhea; 8.3% in the control group had AEs such as anorexia and diarrhea (χ^2^ = 2.33, *p* = 0.33)	NOS, 5	All patients received IFN α2b spray therapy and standard care *
Yan et al. 2020 [34]; China	Retrospective; cohort; single center	Median (IQR), 52 (35–63)	54 (45)	Confirmed COVID-19 cases and had the available RNA viral data to estimate the duration of viral shedding	78 patients received: LPV/RTV (oral): 200/50 mg twice daily for 10 days or more	42 patients received no antiviral therapy (conventional group)	10 days (IQR 7–13)	Median duration of viral shedding was shorter in the LPV/RTV treatment group than that in no LPV/RTV treatment group (median, 22 days vs. 28.5 days, *p* = 0.02) Patients who started LPV/RTV treatment within 10 days from symptom onset had a shorter duration of SARS-CoV-2 RNA shedding than other patients who began after 10 days (median 19 days vs. 27.5 days, *p* < 0.001)	Not reported	NOS, 5	Many patients received and standard care * if in need
Yuan et al. 2020 [35]; China	Retrospective; cohort; single center	Median (range), 40 (1–78)	42 (45)	Confirmed COVID-19 cases of mild and/or moderate symptoms and critical conditions Significant different illness onset on the most common symptoms (fever, fatigue, and diarrhea)	46 patients received: LPV/RTV+ IFN-α (dosages, durations were not reported)	41 patients received: IFN-α + LPV/RTV PLUS Ribavirin; (dosages, durations were not reported)	Not reported	No significant difference in average LOS or PCR negative conversion times among different antivirus treatment groups. Correlation analysis indicated that the duration of hospital stay was significantly correlated with PCR negative conversion times in IFN-α + lopinavir/ritonavir + ribavirin group (*p* = 0.0215), as well as IFN-α + lopinavir/ritonavir group (*p* = 0.012). Average LOS and IFN treatment duration of moderate group was 14.12 (13.34–14.90) days and 14.24 (13.45–15.03) days, respectively, while those of the severe group took average 2.08 days and 1.44 days longer	Not reported	NOS, 6	Approximately 51% were aged ≤40 year, including 2 children under 3 year
Zhu et al. 2020 [36]; China	Retrospective; cohort; multicenter	Mean (SD), 39.8 (17.6)	26 (52)	Confirmed COVID-19 cases No significant difference in age and sex between the two groups	34 patients received: LPV/RTV (oral): 200/50 mg twice daily for 7 days	16 patients received: Umifenovir (oral): 200 mg three times daily (duration was not reported)	Not reported	No difference in fever duration between the two groups (*p* = 0.61). On day 14 after the admission, no viral load was detected in umifenovir group, but the viral load was found in 44.1% of patients treated with LPV/RTV. Patients in the umifenovir group had a shorter duration of positive RNA test compared to those in the LPV/RTV group (*p* < 0.01)	No apparent SEs were found in both groups	NOS, 6	All patients received and standard care * if in need
Ye et al. 2020 [37]; China	Retrospective; cohort; single center	Range (5–68), of which 9 were <30 and 38 were >30	22 (46.8)	Confirmed COVID-19 cases treated with LPV/RTV or not during hospitalization Different age, sex, and baseline demographics in each group	42 patients received: LPV/RTV (oral): 400/100 mg twice daily or 800/200 mg once daily PLUS Umifenovir (oral): 200 mg three times daily PLUS IFN-α1b (aerosol inhalation): 5 million IUs twice daily; (durations of use were not reported)	5 patients received: Umifenovir (oral): 200 mg three times daily PLUS IFN-α1b (aerosol inhalation): 5 million IUs twice daily; (durations of use were not reported)	Not reported	Patients in the LPV/RTV group returned to normal body temperature in a shorter time (test group: 4.8 ± 1.94 days vs. control group: 7.3 ± 1.53 days, *p* = 0.0364) Patients in the LPV/RTV group were able to turn negative in a shorter period of time (LPV/RTV group: 7.8 ± 3.09 days vs. control group: 12.0 ± 0.82 days, *p* = 0.0219)	Increased level of ALT enzyme in the LPV/RTV group	NOS, 5	All patients received and standard care * if in need
Deng et al. 2020 [38]; China	Retrospective; cohort; single center	Mean (SD), 44.6 (15.8)	17 (51.5)	Confirmed COVID-19 cases of adults (≥ 18 years) with laboratory-confirmed COVID-19 without invasive ventilation Baseline clinical, laboratory, and chest CT characteristics were similar between groups	17 patients received: LPV/RTV (oral): 400/100 mg twice daily	16 patients received: LPV/RTV (oral): 400/100 mg twice daily PLUS Umifenovir (oral): 200 mg three times daily (until coronavirus is detected negative by RT-PCR for three times)	Not reported	SARS-CoV-2 could not be detected for 75% of patients’ nasopharyngeal specimens in the combination group after 7 days, compared with 35% in the monotherapy group (*p* < 0.05). After 14 days, 94% in the combination group and 52.9% in the monotherapy group, respectively, SARS-CoV-2 could not be detected (*p* < 0.05) Chest CT scans were improving for 69% of patients in the combination group after seven days, compared with 29% in the monotherapy group (*p* < 0.05)	Elevated levels of bilirubin in patients (68.7%) Digestive upsets, such as mild diarrhea and nausea were reported in patients (43.7%)	NOS, 6	All patients received and standard care * if in need. Authors never stated which therapy group experienced AEs

Abbreviations: AEs, adverse events; ALT, alanine aminotransferase; CI, confidence interval; COVID-19, coronavirus disease 2019; CT, computed tomography; FiO2, fraction of inspired oxygen; FPV, favipiravir; GI, gastrointestinal; HR, hazard ratio; IFN, interferon; IQR, interquartile range; ITT, intention-to-treat; IUs, international units; LOS, length of hospital stay; LPV/RTV, lopinavir/ritonavir; NA, not applicable; NEWS2, National Early Warning Score 2; NHC, National Health Commission of China; NOS, Newcastle Ottawa Scale; PaO2, partial pressure of oxygen; RR, rate ratio; RoB 2, Version 2 of the Cochrane risk-of-bias tool for randomized trials; ROBINS-I, Risk of bias in non-randomized studies—of interventions; RT-PCR, real-time reverse transcription-polymerase chain reaction; SaO2, oxygen saturation; SARS-CoV-2, severe acute respiratory syndrome coronavirus 2; SCI, subcutaneous injection; SEs, side effects. * Standard care comprised, as necessary, supplemental oxygen, non-invasive and invasive ventilation, antibiotic agents, vasopressor support, renal replacement therapy, and extracorporeal membrane oxygenation (ECMO).

## Abbreviations

COVID-19	coronavirus disease 2019
SARS-CoV-2	severe acute respiratory syndrome coronavirus 2
MERS-CoV	Middle East respiratory syndrome coronavirus
LPV/RTV	lopinavir/ritonavir
PRISMA	Preferred Reporting Items for Systematic reviews and Meta-Analysis
RoB 2	Version 2 of the Cochrane risk-of-bias tool for randomized trials
ROBINS-I	Risk of bias in non-randomized studies—of interventions
RT-PCR	real-time reverse transcription-polymerase chain reaction

## References

[1] Yang W., Cao Q., Qin L., Wang X., Cheng Z., Pan A., Dai J., Sun Q., Zhao F., Qu J. (2020). Clinical characteristics and imaging manifestations of the 2019 novel coronavirus disease (COVID-19): A multi-center study in Wenzhou city, Zhejiang, China. J. Infect..

[2] Tobaigy M., Qashqary S., Al-Dahery A., Mujallad A., Hershan M., Kamal N. (2020). Therapeutic management of patients with COVID-19: A systematic review. Infect. Prev. Pract..

[3] Statista (2020). Number of Coronavirus (COVID-19) Clinical Trials for Drugs and Vaccines Worldwide as of November 12, 2020, by Type.

[4] Mehra M.R., Desai S.S., Ruschitzka F., Patel A.N. (2020). Hydroxychloroquine or chloroquine with or without a macrolide for treatment of COVID-19: A multinational registry analysis. Lancet.

[5] Mehra M.R., Ruschitzka F., Patel A.N. (2020). Retraction—Hydroxychloroquine or chloroquine with or without a macrolide for treatment of COVID-19: A multinational registry analysis. Lancet.

[6] (2020). Coronavirus Updates: Trials to Resume of Anti-Viral Touted by Trump.

[7] Cortegiani A., Ippolito M., Ingoglia G., Iozzo P., Giarratano A., Einav S., Update I. (2020). A systematic review on the efficacy and safety of chloroquine/hydroxychloroquine for COVID-19. J. Crit. Care.

[8] Cvetkovic R.S., Goa K.L. (2003). Lopinavir/ritonavir. Drugs.

[9] Alhumaid S., Tobaiqy M., Albagshi M., Alrubaya A., Algharib F., Aldera A., Alali J. (2018). MERS-CoV transmitted from animal-to-human vs MERSCoV transmitted from human-to-human: Comparison of virulence and therapeutic outcomes in a Saudi hospital. Trop. J. Pharm. Res..

[10] Chan J.F.-W., Yao Y., Yeung M.-L., Deng W., Bao L., Jia L., Li F., Xiao C., Gao H., Yu P. (2015). Treatment with lopinavir/ritonavir or interferon-β1b improves outcome of MERS-CoV infection in a nonhuman primate model of common marmoset. J. Infect. Dis..

[11] Chan K., Lai S., Chu C., Tsui E., Tam C., Wong M., Tse M., Que T., Peiris J., Sung J. (2003). Treatment of severe acute respiratory syndrome with lopinavir/ritonavir: A multicentre retrospective matched cohort study. Hong Kong Med. J..

[12] Smolders E.J., Te Brake L.H., Burger D.M. (2020). SARS-CoV-2 and HIV protease inhibitors: Why lopinavir/ritonavir will not work for COVID-19 infection. Antivir. Ther..

[13] Schoergenhofer C., Jilma B., Stimpfl T., Karolyi M., Zoufaly A. (2020). Pharmacokinetics of Lopinavir and Ritonavir in Patients Hospitalized With Coronavirus Disease 2019 (COVID-19). Ann. Intern. Med..

[14] Alhazzani W., Møller M.H., Arabi Y.M., Loeb M., Gong M.N., Fan E., Oczkowski S., Levy M.M., Derde L., Dzierba A. (2020). Surviving Sepsis Campaign: Guidelines on the management of critically ill adults with Coronavirus Disease 2019 (COVID-19). Intensiv. Care Med..

[15] Cao B., Wang Y., Wen D., Liu W., Wang J., Fan G., Ruan L., Song B., Cai Y., Wei M. (2020). A trial of lopinavir–ritonavir in adults hospitalized with severe Covid-19. N. Engl. J. Med..

[16] Massachusetts General Hospital (2020). Massachusetts General Hospital COVID-19 Treatment Guidance.

[17] MoH (2020). Coronavirus Disease 19 (COVID-19) Guidelines, Saudi Arabia.

[18] HPSC (2020). Interim Public Health, Infection Prevention & Control Guidelines on the Prevention and Management of COVID-19 Cases and Outbreaks in Residential Care Facilities in Ireland.

[19] Higgins J.P.T., Thomas J., Chandler J., Cumpston M., Li T., Page M.J., Welch V.A. (2020). Cochrane Handbook for Systematic Reviews of Interventions.

[20] Shamseer L., Moher D., Clarke M., Ghersi D., Liberati A., Petticrew M., Shekelle P., Stewart L.A. (2015). Preferred reporting items for systematic review and meta-analysis protocols (PRISMA-P) 2015: Elaboration and explanation. BMJ.

[21] USFDA (2020). Coronavirus (COVID-19) Update: FDA Revokes Emergency Use Authorization for Chloroquine and Hydroxychloroquine.

[22] Wang Z., Nayfeh T., Tetzlaff J., O’Blenis P., Murad M.H. (2020). Error rates of human reviewers during abstract screening in systematic reviews. PLoS ONE.

[23] Sterne J.A., Savović J., Page M.J., Elbers R.G., Blencowe N.S., Boutron I., Cates C.J., Cheng H.-Y., Corbett M.S., Eldridge S.M. (2019). RoB 2: A revised tool for assessing risk of bias in randomised trials. BMJ.

[24] Sterne J.A., Hernán M.A., Reeves B.C., Savović J., Berkman N.D., Viswanathan M., Henry D., Altman D.G., Ansari M.T., Boutron I. (2016). ROBINS-I: A tool for assessing risk of bias in non-randomised studies of interventions. BMJ.

[25] Wells G.A., Shea B., O’Connell D., Peterson J., Welch V., Losos M., Tugwell P. (2020). Ottawa Hospital Research Institute: The Newcastle-Ottawa Scale (NOS) for Assessing the Quality of Nonrandomised Studies in Meta-Analyses.

[26] Lan X., Shao C., Zeng X., Wu Z., Xu Y. (2020). Lopinavir-ritonavir alone or combined with arbidol in the treatment of 73 hospitalized patients with COVID-19: A pilot retrospective study. MedRxiv.

[27] Li Y., Xie Z., Lin W., Cai W., Wen C., Guan Y., Mo X., Wang J., Wang Y., Peng P. (2020). Efficacy and safety of lopinavir/ritonavir or arbidol in adult patients with mild/moderate COVID-19: An exploratory randomized controlled trial. Med.

[28] Horby P.W., Mafham M., Bell J.L., Linsell L., Staplin N., Emberson J., Palfreeman A., Raw J., Elmahi E., Prudon B. (2020). Lopinavir–ritonavir in patients admitted to hospital with COVID-19 (RECOVERY): A randomised, controlled, open-label, platform trial. Lancet.

[29] Pan H., Peto R., Karim Q.A., Alejandria M., Restrepo A.M.H., García C.H., Kieny M.P., Malekzadeh R., Murthy S., Preziosi M.-P. (2020). Repurposed antiviral drugs for COVID-19; interim WHO SOLIDARITY trial results. MedRxiv.

[30] Cai Q., Yang M., Liu D., Chen J., Shu D., Xia J., Liao X., Gu Y., Cai Q., Yang Y. (2020). Experimental treatment with favipiravir for COVID-19: An open-label control study. Engineering.

[31] Hung I.F.-N., Lung K.-C., Tso E.Y.-K., Liu R., Chung T.W.-H., Chu M.-Y., Ng Y.-Y., Lo J., Chan J., Tam A.R. (2020). Triple combination of interferon beta-1b, lopinavir–ritonavir, and ribavirin in the treatment of patients admitted to hospital with COVID-19: An open-label, randomised, phase 2 trial. Lancet.

[32] Wen C., Xie Z., Li Y., Deng X., Chen X., Cao Y., Ou X., Lin W., Li F., Cai W. (2020). Real-world efficacy and safety of lopinavir/ritonavir and arbidol in treating with COVID-19: An observational cohort study. Zhonghua Nei Ke Za Zhi.

[33] Jun C., Yun L., Xiuhong X., Ping L., Feng L., Tao L., Shang Z., Mei W., Yinzhong S., Hongzhou L. (2020). Efficacies of lopinavir/ritonavir and abidol in the treatment of novel coronavirus pneumonia. Chin. J. Infect. Dis..

[34] Yan D., Liu X.-Y., Zhu Y.-N., Huang L., Dan B.-T., Zhang G.-J., Gao Y.-H. (2020). Factors associated with prolonged viral shedding and impact of Lopinavir/Ritonavir treatment in hospitalised non-critically ill patients with SARS-CoV-2 infection. Eur. Respir. J..

[35] Yuan J., Zou R., Zeng L., Kou S., Lan J., Li X., Liang Y., Ding X., Tan G., Tang S. (2020). The correlation between viral clearance and biochemical outcomes of 94 COVID-19 infected discharged patients. Inflamm. Res..

[36] Zhu Z., Lu Z., Xu T., Chen C., Yang G., Zha T., Lu J., Xue Y. (2020). Arbidol monotherapy is superior to lopinavir/ritonavir in treating COVID-19. J. Infect..

[37] Ye X., Luo Y., Xia S., Sun Q., Ding J., Zhou Y., Chen W., Wang X., Zhang W., Du W. (2020). Clinical efficacy of lopinavir/ritonavir in the treatment of Coronavirus disease 2019. Eur. Rev. Med. Pharm. Sci..

[38] Deng L., Li C., Zeng Q., Liu X., Li X., Zhang H., Hong Z., Xia J. (2020). Arbidol combined with LPV/r versus LPV/r alone against Corona Virus Disease 2019: A retrospective cohort study. J. Infect..

[39] Lian N., Xie H., Lin S., Huang J., Zhao J., Lin Q. (2020). Umifenovir treatment is not associated with improved outcomes in patients with coronavirus disease 2019: A retrospective study. Clin. Microbiol. Infect..

[40] Haviernik J., Štefánik M., Fojtíková M., Kali S., Tordo N., Rudolf I., Hubálek Z., Eyer L., Ruzek D. (2018). Arbidol (Umifenovir): A broad-spectrum antiviral drug that inhibits medically important arthropod-borne flaviviruses. Viruses.

[41] Klement-Frutos E., Burrel S., Peytavin G., Marot S., Lê M.P., Godefroy N., Calvez V., Marcelin A.-G., Caumes E., Pourcher V. (2020). Early administration of ritonavir-boosted lopinavir could prevent severe COVID-19. J. Infect..

[42] Choy K.-T., Wong A.Y.-L., Kaewpreedee P., Sia S.-F., Chen D., Hui K.P.Y., Chu D.K.W., Chan M.C.W., Cheung P.P.-H., Huang X. (2020). Remdesivir, lopinavir, emetine, and homoharringtonine inhibit SARS-CoV-2 replication in vitro. Antivir. Res..

[43] Smith P.F., Dodds M., Bentley D., Yeo K., Rayner C. (2020). Dosing will be a key success factor in repurposing antivirals for COVID-19. Br. J. Clin. Pharmacol..

[44] Fan J., Zhang X., Liu J., Yang Y., Zheng N., Liu Q., Bergman K., Reynolds K., Huang S.-M., Zhu H. (2020). Connecting hydroxychloroquine in vitro antiviral activity to in vivo concentration for prediction of antiviral effect: A critical step in treating COVID-19 patients. Clin. Infect. Dis..

[45] Lamontagne F., Agoritsas T., Macdonald H., Leo Y.-S., Diaz J., Agarwal A., Appiah J.A., Arabi Y., Blumberg L., Calfee C.S. (2020). A living WHO guideline on drugs for covid-19. BMJ.

[46] World Health Organization (2020). Therapeutics and COVID-19: Living Guideline.

[47] National Institutes of Health (2020). Coronavirus Disease 2019 (COVID-19) Treatment Guidelines.

[48] Infectious Diseases Society of America (2020). Guidelines on the Treatment and Management of Patients with COVID-19.

[49] WHO (2020). Draft Landscape of COVID-19 Candidate Vaccines.

[50] Krammer F. (2020). SARS-CoV-2 vaccines in development. Nature.

[51] Moderna, Inc. (2020). Moderna’s COVID-19 Vaccine Candidate Meets Its Primary Efficacy Endpoint in the First Interim Analysis of the Phase 3 COVE Study.

[52] BioNTech P.A. (2020). Pfizer and BioNTech Conclude Phase 3 Study of COVID-19 Vaccine Candidate, Meeting All Primary Efficacy Endpoints.

[53] Halasz S., Fox K., Cassidy A. (2020). AstraZeneca’s Oxford Coronavirus Vaccine is 70% Effective on Average, Data Shows, with No Safety Concerns.

[54] Sheahan T.P., Sims A.C., Leist S.R., Schäfer A., Won J., Brown A.J., Montgomery S.A., Hogg A., Babusis D., Clarke M.O. (2020). Comparative therapeutic efficacy of remdesivir and combination lopinavir, ritonavir, and interferon beta against MERS-CoV. Nat. Commun..

